# Hyper-Activation of mPFC Underlies Specific Traumatic Stress-Induced Sleep–Wake EEG Disturbances

**DOI:** 10.3389/fnins.2020.00883

**Published:** 2020-08-18

**Authors:** Tingting Lou, Jing Ma, Zhiqiang Wang, Yuka Terakoshi, Chia-Ying Lee, Greg Asher, Liqin Cao, Zhiyu Chen, Katsuyasu Sakurai, Qinghua Liu

**Affiliations:** ^1^International Institute for Integrative Sleep Medicine (WPI-IIIS), University of Tsukuba, Tsukuba, Japan; ^2^HIT Center for Life Sciences (HCLS), School of Life Sciences and Technology, Harbin Institute of Technology, Harbin, China; ^3^National Institute of Biological Sciences (NIBS), Beijing, China; ^4^Tsinghua Institute of Multidisciplinary Biomedical Research (TIMBR), Tsinghua University, Beijing, China

**Keywords:** traumatic stress, single prolonged stress (SPS), sleep disturbances, electroencephalogram (EEG), medial prefrontal cortex (mPFC)

## Abstract

Sleep disturbances have been recognized as a core symptom of post-traumatic stress disorders (PTSD). However, the neural basis of PTSD-related sleep disturbances remains unclear. It has been challenging to establish the causality link between a specific brain region and traumatic stress-induced sleep abnormalities. Here, we found that single prolonged stress (SPS) could induce acute changes in sleep/wake duration as well as short- and long-term electroencephalogram (EEG) alterations in the isogenic mouse model. Moreover, the medial prefrontal cortex (mPFC) showed persistent high number of c-fos expressing neurons, of which more than 95% are excitatory neurons, during and immediately after SPS. Chemogenetic inhibition of the prelimbic region of mPFC during SPS could specifically reverse the SPS-induced acute suppression of delta power (1–4 Hz EEG) of non-rapid-eye-movement sleep (NREMS) as well as most of long-term EEG abnormalities. These findings suggest a causality link between hyper-activation of mPFC neurons and traumatic stress-induced specific sleep–wake EEG disturbances.

## Introduction

Exposure to catastrophic traumatic events could lead to severe mental and behavioral disorders, so called post-traumatic stress disorders (PTSD), which are characterized by symptoms of re-experiencing, numbing, avoidance, and hyperarousal (Germain, 2013; Khazaie et al., 2016). Sleep disturbances represent a core symptom of PTSD patients, including insomnia, nightly awakenings, nightmares, sleep paralysis, and restless sleep (Ross et al., 1989; Steine et al., 2012; Khazaie et al., 2016). Although polysomnographic studies in PTSD patients have reported abnormal sleep–wake architecture, previous studies have produced inconsistent results, such as changes in sleep amount, sleep latency, and frequency of nightly awakenings (Kobayashi et al., 2007; Yetkin et al., 2010).

There are also inconsistent results from quantitative analysis of the sleep–wake electroencephalogram (EEG) of PTSD patients (Germain, 2013; Khazaie et al., 2016). Typically, EEG signals can be decomposed into four distinct frequency bands, such as delta (1–4 Hz), theta (5–8 Hz), alpha (9–14 Hz), and beta (15–30 Hz), which may correspond to the underlying intra- and inter-cellular signaling, neuronal activities of different brain regions, brain physiological states, cognitive and mental conditions (Campbell, 2009). For example, there have been reports of increased (Woodward et al., 2000; Insana et al., 2012), decreased (Cohen et al., 2013), or no difference (Mellman et al., 2007) in the beta power of EEG during rapid-eye-movement sleep (REMS) in adult PTSD patients. Both reduced and increased delta power activity during non-rapid-eye-movement sleep (NREMS) and REMS have also been reported in PTSD patients (Woodward et al., 2000; Germain et al., 2006; Insana et al., 2012; de Boer et al., 2020; Wang et al., 2020). These conflicting findings may be attributed to the effects of many confounding variables in the experimental settings, the inter-individual differences and disease heterogeneity, such as differences in initial traumatic stimuli, analysis stages of the illness, comorbidities with other psychiatric conditions, and diversity of underlying neural mechanisms (Kobayashi et al., 2007; Yetkin et al., 2010; Germain, 2013; Baglioni et al., 2016; Khazaie et al., 2016; Deslauriers et al., 2018).

Because PTSD is a heterogeneous disorder affected by many physiological and environmental factors, the development of effective animal models to study traumatic stress-induced sleep abnormalities is urgently needed to fully understand PTSD pathogenesis and pre-clinically evaluate potential treatments (Deslauriers et al., 2018; Mysliwiec et al., 2018). Multiple traumatic stress protocols, such as single prolonged stress (SPS) (Liberzon et al., 1997; Liberzon et al., 1999; Perrine et al., 2016), inescapable foot shocks (Philbert et al., 2011; Yu et al., 2015), restraint stress (Meerlo et al., 2001; Hegde et al., 2008), predator scent stress (Sharma et al., 2018), acute and chronic social defeat stress (Meerlo et al., 1997; Kamphuis et al., 2015; Henderson et al., 2017; Olini et al., 2017; Fujii et al., 2019) have been used to develop PTSD models in rodents. Among these, SPS is a simple and well-established rodent model of traumatic stress that can reliably induce PTSD-like behavioral and physiological abnormalities (Liberzon et al., 1997, 1999; Perrine et al., 2016; Deslauriers et al., 2018).

Although sleep disturbances has long been recognized as a core symptom of PTSD, the PTSD-related sleep phenotypes remain an understudied area. Previously, the effects of SPS on the sleep–wake architecture have only been investigated in two rat studies that yielded inconsistent results (Nedelcovych et al., 2015; Vanderheyden et al., 2015). While one study showed that SPS caused an increase of REMS in the dark phase, but no change in NREMS (Vanderheyden et al., 2015), another reported that SPS reduced both NREMS and REMS in the light phase, followed by a strong rebound in NREMS and REMS in the dark phase (Nedelcovych et al., 2015). These inconsistent results across different laboratories need to be carefully re-examined (Deslauriers et al., 2018).

An important issue that has received little attention is the adopted method for quantitative analysis of the EEG power spectrum. Both absolute and relative EEG power analyses have been commonly used in the literature according to the experimental design (Campbell, 2009; Wang et al., 2018). Absolute EEG power analysis is appropriate for the longitudinal design to measure absolute changes in the EEG power spectrum before and after a traumatic event in the same subject. Relative EEG power analysis, which is calculated as the percentage of power density in a specific frequency bin in the total power of all frequency bins, is suitable for the cross-sectional design to compare the EEG alterations among different subjects. This is because the individual differences in bone thickness, skull resistance and impedance will cause variations in absolute EEG power values (Benninger et al., 1984). Most studies in PTSD patients use the cross-sectional design and, hence, relative EEG power analysis because it is impossible to measure the baseline sleep–wake architecture immediately before a traumatic event in the same individual (Vanderheyden et al., 2015). Some researchers argue that relative EEG power analysis has better test-retest reliability (Salinsky et al., 1991) and sensitivity to age-dependent changes in the frequency composition of EEG signals (Clarke et al., 2001). Based on our previous studies (Funato et al., 2016; Wang et al., 2018), we recognize that relative EEG power analysis, due to the normalization process needed, is likely to miss critical changes of the EEG power spectrum that can be observed from absolute EEG power analysis.

Animal model and clinical studies of PTSD have revealed structural and functional alterations in multiple brain regions, however, the neurological correlates of traumatic stress-induced sleep abnormalities remain largely unexplored (Karl et al., 2006; Deslauriers et al., 2018; Mysliwiec et al., 2018). In particular, it has been challenging to establish the causality link between any specific brain region and traumatic stress-induced sleep abnormalities. In this study, we aim to characterize the SPS mouse model of PTSD, with an emphasis on the sleep–wake phenotypes. We found that SPS-treated mice exhibited specific changes in the sleep–wake architecture, including both short- and long-term EEG alterations. Moreover, our results suggest for the first time a causality link between the hyper-activation of medial prefrontal cortex (mPFC) neurons and the SPS-induced specific sleep–wake EEG abnormalities. This type of investigations should be important to understanding the neural mechanisms and facilitating development of effective therapies for at least a subset of PTSD patients.

## Materials and Methods

### Animal Subjects

All mice were housed under humidity and temperature (22–25±°C) controlled conditions on a 12-h light–dark cycle with food and water provided *ad libitum*. We used 12–20 weeks old (26–33 g body weight) C57BL/6N male mice (CLEA Japan) in this study. All experimental animal procedures were approved by the Institutional Animal Care and Use Committee of University of Tsukuba. All mice were singly housed for one week before each experiment.

### Sleep Deprivation and Single Prolonged Stress

For sleep deprivation, mice were sleep deprived for 4 h from the onset of the light phase (ZT0–ZT4) by gently touching the cages when they started to recline and lower their heads in the home cage. The SPS was performed at the onset of light phase (ZT0) as previously described (Liberzon et al., 1997, 1999; Deslauriers et al., 2018). First, each mouse was restrained for 2 h in a 50 ml Falcon tube with the bottom removed. Second, the mouse was forced to swim for 20 min in a plastic cylinder (height: 25 cm; diameter: 18.5 cm) filled with water (21–24°C), such that the mouse’s hind limbs could not touch the bottom. Third, after recuperating for 15 min in a new cage, the mouse was exposed to ether until general anesthesia (no more than 5 min). Finally, the mouse was returned to its home cage (around ZT3.5) and sleep deprived until ZT4 by gently touching the cages.

### EEG/EMG Electrode Implantation

Mice (8–10 weeks old) were implanted with the EEG/electromyogram (EMG) electrodes under anesthesia by isoflurane (3% for induction and 1% for maintenance). Briefly, four electrode pins were lowered to the dura under stereotaxic control. Two electrodes for EEG signals were positioned over the frontal and occipital cortices [anteroposterior (AP): 0.5 mm, mediolateral (ML): 1.3 mm, dorsoventral (DV): −1.3 mm; and AP: −4.5 mm, ML: 1.3 mm, DV: −1.3 mm]. Two electrodes with flexible wires for EMG recording were threaded through the dorsal neck muscle. Afterward, the EEG/EMG electrodes were glued to the skull with dental cement. Mice were individually housed following surgery, and allowed a minimum recovery period of 7 days.

### Sleep–Wake Behaviors Analysis

The sleep–wake behaviors were analyzed as previously described (Funato et al., 2016; Wang et al., 2018). Mice were tethered to a counterbalanced arm (Instech Laboratories) that allowed free movement and exerted minimal weight, and acclimatized to the recording chamber around 7 days before recording. EEG/EMG signals were recorded at the age of 12–20 weeks; age-matched animals were used in each experiment for control and treatment groups. EEG/EMG data were analyzed using a MatLab (MathWorks)-based semi-automated staging software followed by manual correction. EEG signals were decomposed by fast Fourier transform analysis for 1 to 30 Hz with 1 Hz bins. Sleep/wake states were scored in 20 s epoch as wake (low amplitude, fast EEG and high amplitude, and variable EMG), REMS [dominant theta (5–8 Hz) EEG and EMG atonia], or NREMS [high amplitude delta (1–4 Hz) EEG and low EMG tonus]. Absolute (arbitrary units) and relative EEG power density analysis was performed to examine the delta (1–4 Hz), theta (5–8 Hz), alpha (9–14 Hz), and beta (15–30 Hz) activities during NREMS, REMS, or wake state at indicated ZT period. To minimize the inter-individual differences for following statistical analysis, absolute EEG power data of each individual animal for the corresponding NREMS, REMS, or wake state was normalized to the mean power from ZT8 to ZT11 of baseline recording day of all animals used within each corresponding experiment, which is at the end of the major rest period (Franken et al., 2001; Mang et al., 2016). Relative EEG power density analysis (%) is defined by the ratio of a specific frequency bin to the total power over all frequency bins (1–30 Hz). In hourly analysis of sleep–wake architecture, each data point represents the mean value of either duration or EEG power density in the following 1 h during NREMS, REMS, and wake states. Researchers were blinded to genotype and/or treatment before data analysis, and only animals with unreadable EEG signals were excluded from final analysis.

### Behavioral Experiments

Two groups of mice were sleep deprived for 4 h (SD4) or exposed to SPS treatment, respectively. On the 7th day after the SD4/SPS procedure, the tail suspension test (TST) was performed as previously described (Can et al., 2012b). Each mouse was suspended in the hook of an open front TST box, approximately 50 cm above the surface of table with a small piece of adhesive tape placed 2 cm away from the tip of the tail. The duration of immobility was recorded for 10 min by a video camera positioned in front of the test box. Mice were considered immobile only when they hung passively and were completely motionless. Mice were returned to their home cages to rest for at least 1 h, and then the forced swim test (FST) was performed as previously described (Can et al., 2012a). The mice were placed individually for 10 min in a plastic cylinder (height: 25 cm; diameter: 18.5 cm) filled with water (21–24°C) to a depth of 14 cm. The water depth was adjusted so that the animal’s hind limbs cannot touch the bottom. Water was changed between subjects. All test sessions were recorded by a video camera positioned on the top of the plastic cylinder. Mice were considered to be immobile when floating motionless or making only those movements necessary to keep its head above the water. The duration of immobility was measured manually by an observer blind to group assignment.

### Immunohistochemistry

All mice were singly housed at least for 1 week before experiments. After experimental treatments, test mice were allowed to recover in the home cage. Specifically, at least one paired control and stressed mice brains were harvested at 30 min (ZT4.5) or 3.5 h (ZT7.5) after SPS or SD4 treatment at the same experimental day and processed at the same time in the following steps. At indicated ZT time, paired control and stressed mice were rapidly anesthetized with pentobarbital (50 mg/kg, i.p.), and then transcardially perfused with 0.1 M phosphate buffer saline, pH7.4 (PBS), followed by 4% paraformaldehyde in PBS (PFA). Whole brain was dissected and post-fixed for 24-h in 4% PFA at 4°C, and then cryoprotected with 30% sucrose (wt/vol) in PBS for 48 h at 4°C. The tissues were frozen in the Tissue-Tek O.C.T compound (Sakura Finetek), and 80-μm-thick coronal sections were cut on a cryostat (CM3050S, Leica). For c-Fos staining, the floating brain sections were washed three times with PBS for 5 min each, incubated with 1% Triton X-100 in PBS for 2 h. The sections were incubated in 10% Blocking One (nacalai tesque) in PBS with 0.1% Triton-X-100 (blocking solution) for 1-h at room temperature. The sections were incubated with rabbit anti-c-Fos antibody (1:2,500, EMD Millipore, ABE457) in blocking solution at 4°C overnight. After washing three times with PBS, the sections were incubated with Donkey anti-rabbit Alex488 (1:500, Thermo Fisher R37118) and Fluorescent Nissl Stain (1:500, Thermo Fisher N21479) in Blocking solution at 4°C overnight. After washing three times with PBS, the sections were mounted and covered with coverslip. All images were acquired using the Zeiss LSM700 confocal microscope with a 10× objective lens (NA = 0.45) under the Zen 2010 software (Carl Zeiss). The c-Fos positive neurons were counted in all sections from the same mouse brain (ImageJ). No normalization was performed for the c-fos expression at ZT4.5 and ZT7.5. Representative images shown in the figures were chosen from a similar region based on morphology.

### *In situ* Hybridization

The cDNA fragments of mouse *c-fos*, *vGlut1*, and *vGat* were amplified by PCR with antisense primers containing T3 or T7 promoter sequence. *In vitro* transcription was performed with PCR-amplified template using T3 RNA polymerase (Promega) or T7 RNA polymerase (Roche) for the synthesis of antisense probes. Fluorescent two-color *in situ* hybridization was performed based on a basic method (Ishii et al., 2017). Briefly, mice were subjected to SPS treatment and, after 30 min, were anesthetized with pentobarbital (50 mg/kg, i.p.) followed by perfusion with 4% paraformaldehyde (PFA) in PBS. Brain slices (40 μm) were treated with protease K (Roche, cat#03115887001), followed by acetylation. The brain slices were incubated with hybridization buffer containing RNA probe mix at 60°C for 16 h. After stringent washing, brain slices were incubated with horseradish peroxidase (HRP) conjugated anti-FITC antibody (PerkinElmer, 1:1,000) or HRP-conjugated anti-Dig antibody (Roche; 1:1,000) overnight at 4°C. TSA system (TSA-FITC or TSA-Biotin; PerkinElmer) was applied to visualize the mRNA signal. All images were acquired using the Zeiss LSM700 confocal microscope with a 10× objective lens (NA = 0.45) under the Zen 2010 software (Carl Zeiss). The *c-fos*, *vGlut1*, and *vGat* positive neurons were counted in all sections from the same mouse brain (Image J).

### Stereotaxic AAV Injection and Drug Administration

For bilateral injection of adeno-associated viruses (AAV) (AAV2/9-CMV-mCherry; AAV2/9-hSyn-hM4Di–mCherry) into the mPFC, male mice (8–10 weeks old) were anesthetized with isoflurane (3% for induction and 1% for maintenance) and placed in a stereotaxic frame (David Kopf Instruments). An incision was made on the top of the skull, and the skin was retracted and connective tissue gently scraped away. After exposing the skull and cleaning the surface with 3% hydrogen peroxide, bilateral craniotomies (∼1 mm diameter each) were made to allow virus delivery (500 nl at 100 nl/min). Stereotaxic coordinates of virus injection were based on Paxinos and Franklin mouse brain atlas (AP: −1.94 mm, L: ±0.4 mm, DV: −2 mm). For EEG/EMG analysis of AAV-injected mice, the EEG/EMG electrode implantation was performed immediately following AAV injection. Clozapine N-oxide (CNO; Cayman Chemical, Item No. 12059) was dissolved in saline. Vehicle (0.9% saline) or CNO (3 mg/kg) was administered by intraperitoneal injection at ZT0 and ZT3.5 before the mouse returned to the home cage.

### Statistical Methods

GraphPad Prism 6 software was used for statistical tests. No statistical method was used to predetermine sample size. Randomization was not used. Following two-way ANOVA analysis of variance, Sidak’s test was performed to compare a set of means, repeated measures was performed for matched subject comparisons. Paired *t*-test was performed for matched subject comparisons, whereas unpaired *t*-test for group comparisons. The complete sample size, statistical test method and results for each comparison are reported in the figure legends and described in detail in Supplementary Table 1. *P* < 0.05 was considered statistically significant. Unless otherwise noted, all experimental subjects are biological replicates and at least two independent experiments were performed.

## Results

**FIGURE 1:**
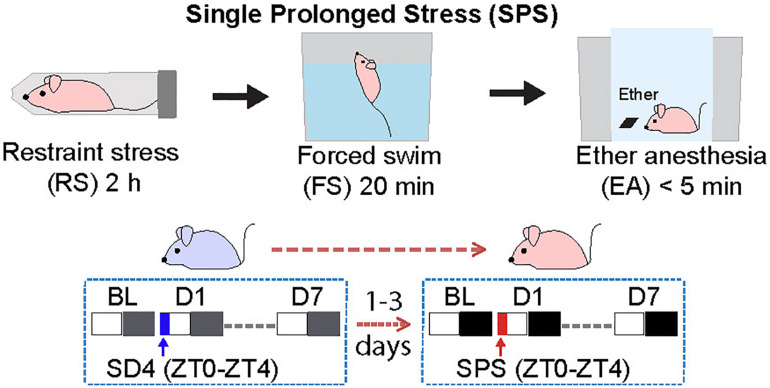
Experimental design for studying single prolonged stress (SPS)-induced sleep–wake disturbances. The same group of C57BL/6N male mice (*n* = 20) were subjected to seven day EEG/EMG recording after sleep deprivation (SD4, ZT0–ZT4), and followed by seven day EEG/EMG recording after single prolonged stress (SPS, ZT0–ZT4).

We adopted the standardized SPS paradigm to investigate the effects of traumatic stress on the sleep–wake architecture in wild-type C57BL/6N male mice. We used a longitudinal experimental design by sequentially comparing sleep/wake changes before and after 4-h sleep deprivation by gentle handling (SD4, ZT0–ZT4) or SPS (ZT0–ZT4) on the same subjects (Figure 1). For the SD4 segment, after continuous 24-h baseline (SD4-BL) recording, all test mice are subjected to SD4 (SD4-D1) and continuously monitored for EEG and EMG in the home cage until the seventh day (SD4-D7). After 1–3 days’ rest, the same mice would be subjected to SPS (ZT0–ZT4) to study how traumatic stress caused sleep–wake disturbances. For the SPS segment, after continuous 24 h baseline (SPS-BL) recording, all test mice were subjected sequentially to 2-h restraining, 20-min forced swimming, and up to 5-min anesthesia by ether (SPS-D1), and followed by continuous EEG/EMG recording until the 7th day (SPS-D7). This longitude design gave us two important advantages over previous studies: (a) comparison of SPS and SD4 could distinguish the specific effects of SPS (as opposed to prolonged wakefulness) on the sleep–wake architecture; (b) the baseline and post-SD4 or post-SPS EEG/EMG recordings of the same mice allowed for both absolute and relative EEG power analysis to comprehensively evaluate the SPS-induced short-term (D1) and long-term (D7) EEG abnormalities, which is not possible in previous SPS rat studies (Nedelcovych et al., 2015; Vanderheyden et al., 2015).

### Traumatic Stress Induces Acute Changes in Sleep/Wake Duration

**FIGURE 2:**
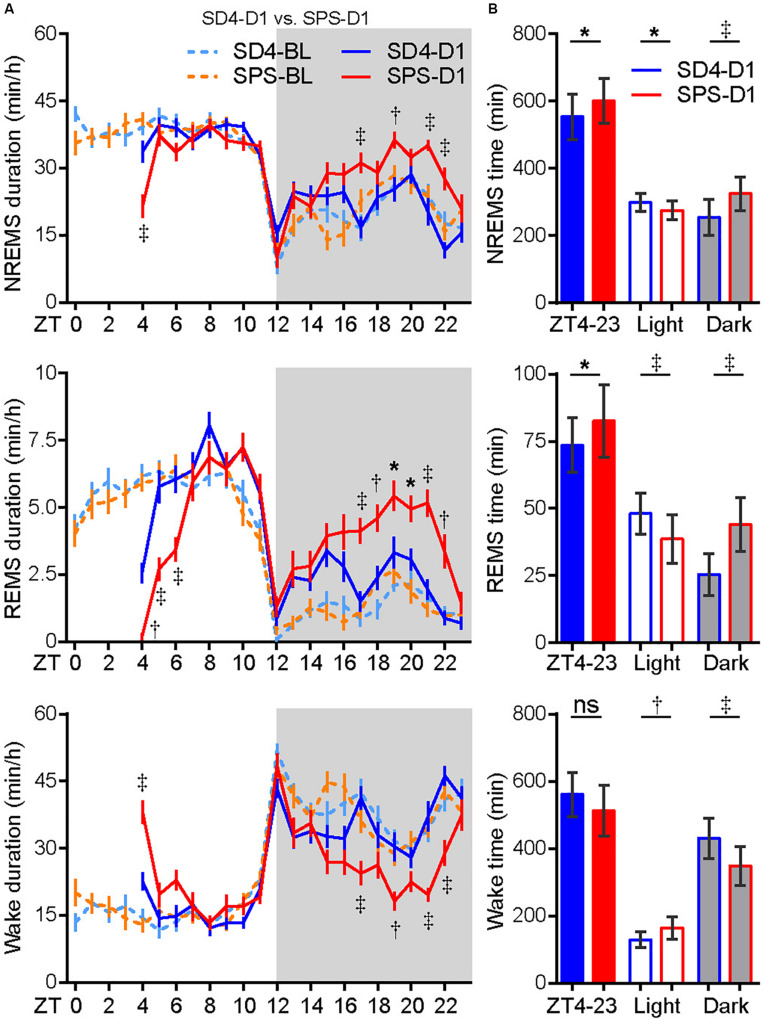
Traumatic stress induces acute changes in sleep/wake duration. **(A)** Analysis of NREMS, REMS, or wake duration in every hour on the day before (SD4-BL and SPS-BL) or after (SD4-D1 and SPS-D1) SD4/SPS treatment. **(B)** Quantitative analysis of total NREMS, REMS, or wake time on the day after SD4 or SPS treatment (SD4-D1 vs SPS-D1). Mean ± s.e.m., two-way ANOVA, Sidak’s test **(A)**; Mean ± s.d., paired *t*-test, two-tailed **(B)**. *P < 0.05; ^†^*P* < 0.01; ^‡^*P* < 0.001; ^ns^*P* > 0.05.

To examine the acute effect of traumatic stress on the sleep–wake architecture, we compared the EEG/EMG data of test mice on the day before (SD4-BL or SPS-BL) and after SD4/SPS (SD4-D1 or SPS-D1) (Figure 2). It is important to note that there were essentially no difference in the baseline sleep–wake pattern of the same mice before SD4 and SPS treatment (SD4-BL vs SPS-BL), making it possible to directly compare the effects of SPS and SD4 on the sleep–wake architecture (Figure 2A, Supplementary Figure 1A and Table 1). On the day after SD4, there was on average a 58.1% reduction in REMS duration (SD4-D1, 2.6 ± 1.9 min vs SD4-D0, 6.2 ± 1.9 min) at the first hour (ZT4) after sleep deprivation, and a 72.3 and 8.2% rebound of REMS (SD4-D1, 25.5 ± 7.8 min vs SD4-D0, 14.8 ± 6.2 min) and NREMS (SD4-D1, 254.2 ± 53.6 min vs SD4-D0, 234.9 ± 50.6 min) in the dark phase, respectively (Figure 2A and Supplementary Figure 1B). On the day after SPS, there was on average a 96.6 and 47.5% reduction in REMS duration (SPS-D1, 0.2 ± 0.6 min vs SPS-BL, 5.9 ± 1.5 min) and NREMS duration (SPS-D1, 21.5 ± 11.2 min vs SPS-BL, 41.0 ± 6.7 min) at the first hour (ZT4) after SPS, as well as a 183.9 and 33.9% rebound of REMS (SPS-D1, 44.0 ± 10.1 min vs SPS-BL, 15.5 ± 7.3 min) and NREMS (SPS-D1, 325.4 ± 49.5 min vs SPS-BL, 243.1 ± 37.6 min) in the dark phase, respectively (Figure 2A and Supplementary Figure 1C).

By direct comparison of the SPS-D1 vs SD4-D1 data, we found that SPS, relative to SD4, resulted in about 36.2% less NREMS at ZT4 (SPS-D1, 21.5 ± 11.2 min vs SD4-D1, 33.7 ± 10.9 min) and 56.3% less REMS during ZT4-6 (SPS-D1, 6.3 ± 3.1 min vs SD4-D1, 14.4 ± 4.0 min) (Figure 2A). In the dark phase, SPS mice spent 28 and 72.5% more time than SD4 mice in NREMS (SPS-D1, 325.4 ± 49.5 min vs SD4-D1, 254.2 ± 53.6 min) and REMS (SPS-D1, 44.0 ± 10.1 min vs SD4-D1, 25.5 ± 7.8 min), respectively (Figure 2B). Thus, our results indicate that traumatic stress by SPS can induce specific changes in the sleep–wake architecture that are distinct from sleep deprivation.

### Traumatic Stress Induces Short-Term Sleep/Wake EEG Abnormalities

**FIGURE 3:**
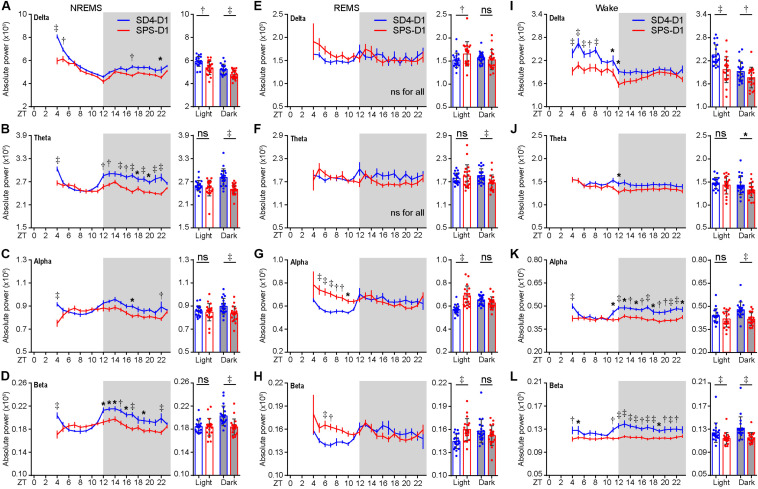
Traumatic stress induces acute changes in sleep/wake EEG power spectrum. **(A–L)** Analysis of mean absolute EEG power density in every hour (left, hourly) or in the light/dark phase (right) during NREMS **(A–D)**, REMS **(E–H)**, and wake **(I–L)** states of test mice (*n* = 20) on the day after SD4/SPS treatment (SD4-D1 vs SPS-D1). Mean ± s.e.m., two-way ANOVA, Sidak’s test (for hourly analysis); Mean ± s.d., paired *t*-test, two-tailed (for mean analysis). *P < 0.05; ^†^*P* < 0.01; ^‡^*P* < 0.001; ^ns^*P* > 0.05.

Similarly, the baseline sleep/wake EEG power spectrum of the test mice was essentially the same before SD4 and SPS treatment (Supplementary Figure 2; SD4-BL vs SPS-BL). By absolute EEG power analysis, SD4 resulted in a broad increase over baseline in all frequency bands of EEG signals during NREMS in the light phase, particularly in the first hour (ZT4) after sleep deprivation (↑56.4% delta; ↑24.3% theta; ↑15.1% alpha; ↑19.0% beta) (Supplementary Figure 3; SD4-D1 vs SD4-BL). On the other hand, SPS resulted in a 13.2 and 9.5% increase over baseline, respectively, in the delta and theta power of EEG signals during NREMS at ZT4 (Supplementary Figure 3A; SPS-D1 vs SPS-BL). Comparison of the SPS and SD4 data reveals that SPS, relative to SD4, caused a broad suppression in all frequency bands of EEG signals during NREMS at ZT4 (↓26.1% delta; ↓12.1% theta; ↓18.0% alpha; ↓16.2% beta) and in the dark phase (↓9.0% delta; ↓11.0% theta; ↓7.2% alpha; ↓9.4% beta), as well as a specific suppression (↓9.4%) of NREMS delta power, a measurable index of sleep need, in the light phase (Figures 3A–D; SPS-D1 vs SD4-D1).

During REMS, SPS, relative to SD4, causes a significant increase in the absolute delta (↑8.8%), alpha (↑20.6%), and beta (↑10.6%) EEG power in the light phase, as well as a 10% reduction in theta EEG power in the dark phase (Figures 3E–H; SPS-D1 vs SD4-D1). During wakefulness, SPS, relative to SD4, caused a significant decrease in absolute delta (↓16.3%) and beta (↓7.9%) power in the light phase (Figures 3I–L; SPS-D1 vs SD4-D1). Additionally, SPS mice exhibited a broad reduction in all frequency bands of EEG signals (↓8.1% delta; ↓7.5% theta; ↓12.8% alpha; ↓12.6% beta) in the dark phase (Figures 3I–L; SPS-D1 vs SD4-D1). These observations indicate that SPS causes specific short-term sleep/wake EEG abnormalities.

### Traumatic Stress Induces Long-Term Sleep/Wake EEG Abnormalities

**FIGURE 4:**
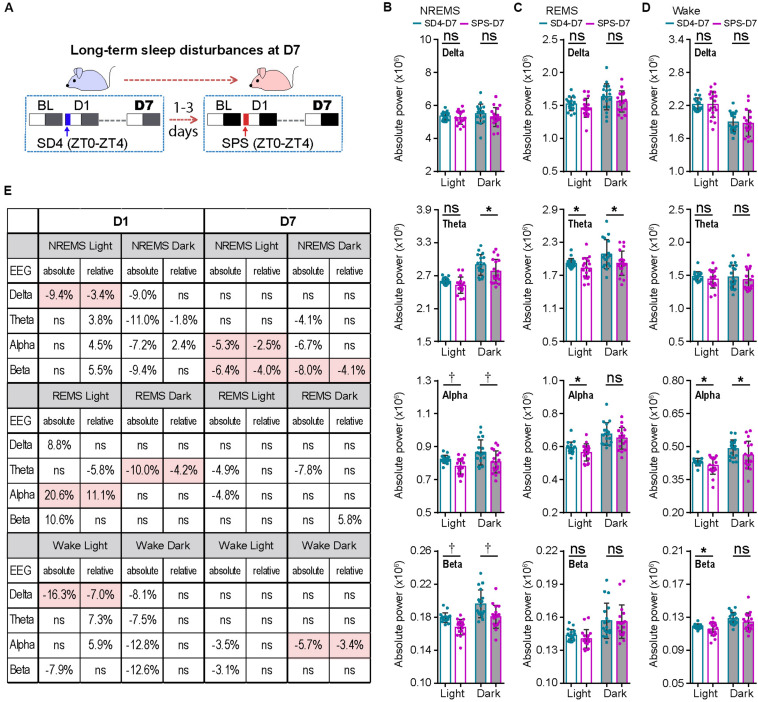
Traumatic stress induces long-term alterations of sleep/wake EEG power spectrum. **(A)** A schematic of sleep–wake analysis at D7 after SD4/SPS treatment. **(B–D)** Analysis of mean absolute EEG power density in NREMS **(B)**, REMS **(C)**, and wake **(D)** states of the same test mice on day 7 after SD4/SPS treatment (SD4-D7 vs SPS-D7). **(E)** A table comparing the specific change ratios of the delta, theta, alpha, and beta power bands of EEG signals detected by absolute and relative EEG power analysis on D1 [(“SPS-D1” – “SD4-D1”)/“SD4-D1”] and on D7 [(“SPS-D7” – “SD4-D7”)/“SD4-D7”] after SD4/SPS treatment. Mean ± s.d., paired *t*-test, two-tailed **(B–D)**. *P < 0.05; ^†^*P* < 0.01; ^ns^*P* > 0.05.

To examine the long-term effect of SPS on sleep–wake architecture, we compared the EEG/EMG data of the same mice on the seventh day (D7) after SD4 and SPS treatment (Figure 4A; SD4-D7 vs SPS-D7). Consistent with the previous study of SPS rats (Nedelcovych et al., 2015), there was no significant difference in the total duration, episode duration, or episode number of NREMS, REMS and wakefulness on D7 after SPS (Supplementary Figures 4A–D). By contrast, SPS, relative to SD4, caused a broad reduction in sleep/wake EEG power densities in the light phase, including the alpha (↓5.3%) and beta (↓6.4%) power during NREMS; the theta (↓4.9%) and alpha (↓4.8%) power during REMS; the alpha (↓3.5%) and beta (↓3.1%) power during wakefulness (Figures 4B–D; SPS-D7 vs SD4-D7). In the dark phase, SPS caused a specific decrease in absolute theta (↓4.1%), alpha (↓6.7%), and beta (↓8.0%) power during NREMS; theta (↓7.8%) power during REMS; and alpha (↓5.7%) power during wakefulness (Figures 4B–D; SPS-D7 vs SD4-D7). Taken together, these observations indicate that unlike sleep deprivation, traumatic stress by SPS can lead to long-term sleep/wake EEG abnormalities.

### Absolute EEG Power Analysis Is Superior to Relative EEG Power Analysis

Our previous studies suggest that relative EEG power analysis is likely to miss critical changes of the EEG signals, such as a global reduction in EEG power densities, which can be detected by absolute EEG power analysis (Funato et al., 2016; Wang et al., 2018). Accordingly, we obtained very different outcomes by relative EEG power analysis (Figure 4E). During NREMS, SPS, relative to SD4, causes variable changes in the EEG power spectrum in the light phase (delta, ↓3.4%; theta, ↑3.8%; alpha,↑4.5%; beta, ↑5.5%) and in the dark phase (delta, ns; theta, ↓1.8%; alpha, ↑2.4%; beta, ns). Remarkably, only the modest reduction in NREMS delta (↓3.4%) power is verified by absolute EEG power analysis (Figure 4E; SPS-D1 vs SD4-D1). During REMS, relative EEG power analysis also reveals variable changes of the EEG power spectrum in the light phase (delta, ns; theta, ↓5.8%; alpha, ↑11.1%; beta, ns) and in the dark phase (delta, ns; theta, ↓4.2%; alpha, ns; beta, ns). Among these changes, only the 11.1% alpha power increase in the light phase and 4.2% theta power reduction in the dark phase are consistent with absolute EEG power analysis (Figure 4E; SPS-D1 vs SD4-D1).

For the long-term sleep/wake EEG abnormalities, relative EEG power analysis reveals that SPS, relative to SD4, causes a modest reduction in the alpha (↓2.5%) and beta (↓4.0%) power of NREMS in the light phase, beta (↓4.1%) power of NREMS in the dark phase, and alpha (↓3.4%) power of wake in the dark phase. Although these changes are largely consistent with those of absolute EEG power analysis, relative EEG power analysis failed to detect many critical changes of the EEG power spectrum in the dark phase (Figure 4E; SPS-D7 vs SD4-D7). Based on these observations, we conclude that absolute EEG power analysis is superior to relative EEG power analysis, which should be adopted especially in the longitude experimental setting.

### Persistent Activation of mPFC Neurons During and After SPS Treatment

**FIGURE 5:**
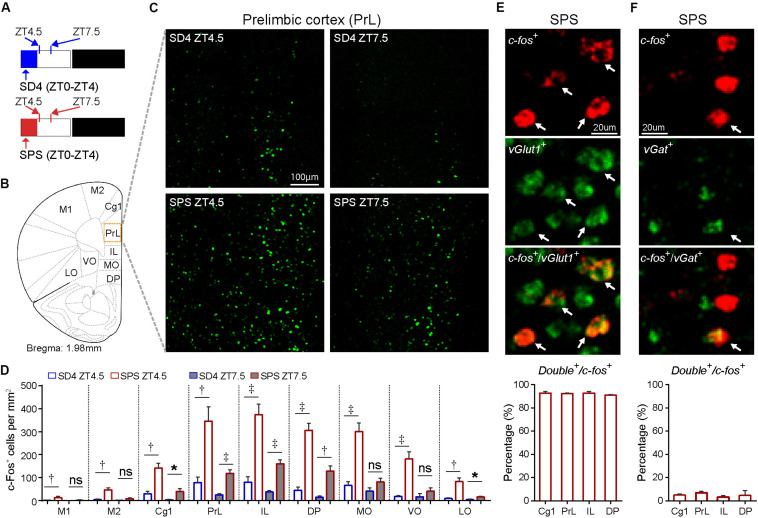
Traumatic stress induces persistent c-Fos expression in the mPFC neurons. **(A)** Experimental design for mapping brain regions showing abnormal c-Fos expression after SPS relative to SD4 treatment. Mouse brains were harvested at 30 min (ZT4.5) or 3.5 h (ZT7.5) after SPS or SD4 treatment. **(B)** A schematic map of mouse prefrontal cortex showing different sub-regions. **(C)** Representative images showing c-Fos immunohistochemistry in the prelimbic cortex (PrL) of SPS/SD4 mice at ZT4.5 and ZT7.5. **(D)** Quantitative analysis of c-Fos–expressing neurons at ZT4.5 and ZT7.5 after SPS/SD4 treatment in different subregions of prefrontal cortex (*n* = 5). Mean ± s.e.m., unpaired *t*-test, two-tailed. **(E,F)** Fluorescent two-color *in situ* hybridization staining and quantitation of *vGlut1*^+^ **(E)** or *vGat*^+^ **(F)**, and *c-fos*^+^ double positive neurons after SPS treatment. *vGlut1*, *n* = 3; *vGat*, *n* = 4. Mean ± s.e.m. Primary (M1) and secondary (M2) cortex of motor cortex; cingulate (Cg1), prelimbic (PrL), infralimbic (IL), and dorsal peduncular (DP) cortex within mPFC; medial (MO), ventral (VO), and lateral (LO) part of the orbitofrontal cortex (OFC). *P < 0.05; ^†^*P* < 0.01; ^‡^*P* < 0.001; ^ns^*P* > 0.05.

Accumulating studies suggest that PTSD may be mediated by structural and functional alterations in multiple brain regions, including the prefrontal cortex, locus coeruleus, amygdala, hippocampus, and the hypothalamic–pituitary–adrenal (HPA) axis (Lindauer et al., 2004; Wignall et al., 2004; Lindauer et al., 2006; Chen et al., 2018; Deslauriers et al., 2018; Logue et al., 2018; Naegeli et al., 2018; van Rooij et al., 2018; Heyn et al., 2019). To explore the neurobiological correlates of traumatic stress-induced sleep abnormalities, we performed comparative analysis of the expression of immediate early gene c-Fos by immunostaining of mouse brain samples harvested at ZT4.5 and ZT7.5 after SD4/SPS treatment (ZT0–ZT4) (Figure 5A). Because c-Fos proteins exhibit a half-life of 45 min for fast decay and 1.5–2 h for slow decay (Shah and Tyagi, 2013), we wanted to identify specific brain regions showing persistent hyper-activity in response to SPS. At ZT4.5, SPS mice, relative to SD4 mice, showed significantly more c-Fos-expressing neurons in multiple subregions of the prefrontal cortex (Figures 5B–D), including the primary (M1) and secondary (M2) cortex of motor cortex (MC), the cingulate (Cg1), prelimbic (PrL), infralimbic (IL), and dorsal peduncular (DP) cortex within the mPFC, and the medial (MO), ventral (VO), and lateral (LO) part of the orbitofrontal cortex (OFC) (Figure 5D). By two-color fluoresence *in situ* hybridization, we showed that more than 95% of *c-fos* positive neurons in the mPFC express the excitatory neuron marker *vGlut1*, but not the inhibitory neuron marker *vGat* (Figures 5E,F). Moreover, while SD4-induced c-Fos expression dissipated, SPS-induced c-Fos expression could still be observed in the mPFC, most notably in the PrL, IL and DP at ZT7.5 (Figures 5C,D). These results suggest SPS causes persistent hyper-activities of mPFC neurons during and immediately after SPS treatment.

### Chemogenetic Inhibition of mPFC Reverses SPS-Induced Sleep/Wake EEG Disturbances

**FIGURE 6:**
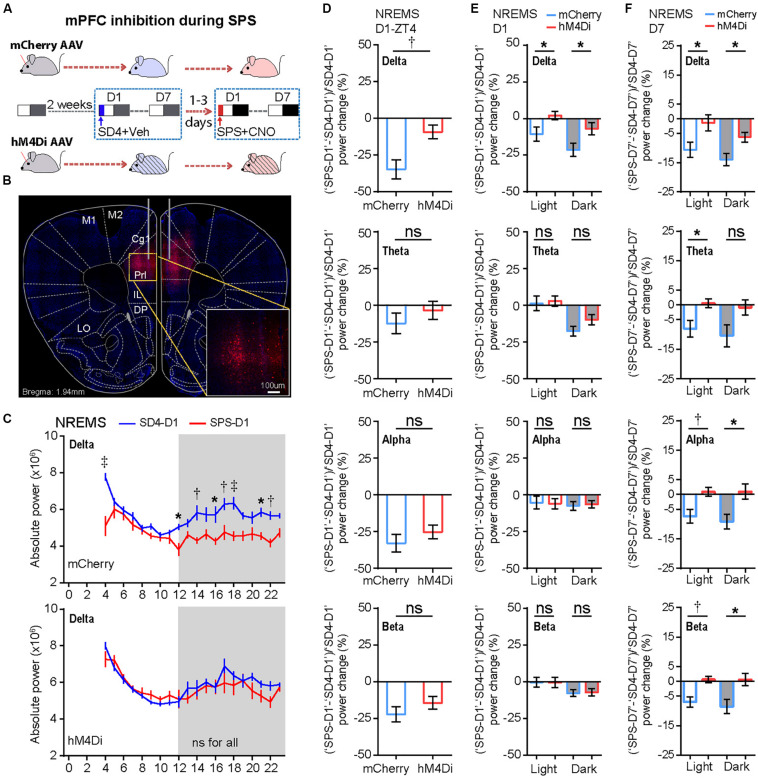
Chemogenetic inhibition of mPFC-PrL neurons specifically reverses SPS-induced acute suppression of NREMS delta power and long-term EEG alterations. **(A)** Experimental design for chemogenetic inhibition of mPFC-PrL during SPS treatment. Two groups of C57BL/6N male mice (*n* = 9) were bilaterally injected into the PrL region of mPFC with AAV2/9-mCherry (mCherry) or AAV2/9-hM4Di-mCherry (hM4Di), respectively. All mice were subjected to seven day EEG/EMG recording after sleep deprivation (SD4, ZT0–ZT4), and subsequently subjected to seven day EEG/EMG recording after SPS (ZT0–ZT4). Intraperitoneal injection of vehicle (0.9% saline) during SD4 or CNO (3 mg/kg) during SPS was administered at ZT0 and ZT3.5. **(B)** Representative image showing correct AAV injection sites marked by mCherry positive cells. **(C)** Hourly analysis of mean absolute delta power density of NREMS in mCherry (*n* = 9) and hM4Di (*n* = 9) mice on the day after SD4/SPS treatment (SD4-D1 vs SPS-D1). **(D,E)** Comparison of the change ratio [(“SPS-D1” – “SD4-D1”)/“SD4-D1”]% in the mean absolute NREMS EEG power density of mCherry and hM4Di mice at ZT4 **(D)**, and in the light or dark phase **(E)**. **(F)** Comparison of the change ratio [(“SPS-D7” – “SD4-D7”)/“SD4-D7”]% in the mean absolute NREMS EEG power density of mCherry and hM4Di mice in the light or dark phase on day 7 after SD4/SPS treatment. Mean ± s.e.m., two-way ANOVA, Sidak’s test **(C)**; Mean ± s.e.m., unpaired *t*-test, two-tailed **(D–F)**. *P < 0.05; ^†^*P* < 0.01; ^‡^*P* < 0.001; ^ns^*P* > 0.05.

We hypothesized that the persistent hyper-activities of mPFC could contribute to the SPS-induced short- and long-term alterations in the sleep–wake architecture and EEG power spectrum. The prelimbic (PrL) region of mPFC is a major subregion to control neuroendocrine outputs of the paraventricular hypothalamic nucleus (PVH) to restore homeostasis of the HPA axis-the central stress response system (Radley et al., 2006; Herman et al., 2012). Therefore, we used the inhibitory Designer Receptors Exclusively Activated by Designer Drugs (DREADD) system to investigate whether hyper-activation of PrL neurons play an important role in traumatic stress-induced sleep–wake disturbances. Specifically, we bilaterally injected AAV expressing mCherry (AAV2/9-CMV-mCherry) or hM4Di (AAV2/9-hSyn-hM4Di-mCherry) into the PrL of mPFC in C57BL/6N mice (Figures 6A,B). All AAV-injected mice were sequentially subjected to SD4 and SPS treatments as described above (Figure 1), except for intraperitoneal injection of vehicle during SD4 or CNO during SPS at ZT0 and ZT3.5, and followed by continuous EEG/EMG recording for seven days (Figure 6A).

We found that chemogenetic inhibition of PrL during SPS could not rescue the SPS-induced acute changes in sleep/wake duration on day 1 (Supplementary Figure 5; “SPS-D1” − “SD4-D1”: mCherry vs hM4Di). However, inhibition of PrL activity could specifically reverse the SPS-induced acute suppression of NREMS delta power [Figures 6C–E and Supplementary Figure 6A; (“SPS-D1” − “SD4-D1”)/“SD4-D1”: mCherry vs hM4Di], particularly in the first hour (ZT4) after SPS (Figures 6C,D). By contrast, there were no statistically significant differences in other EEG power densities during NREMS, REMS or wake states between mCherry and hM4Di mice (Supplementary Figure 6). These results are consistent with the idea that hyper-activities of PrL neurons could result in specific suppression of the NREMS delta power, the best known measurable index of sleep need, immediately after traumatic stress.

**FIGURE 7:**
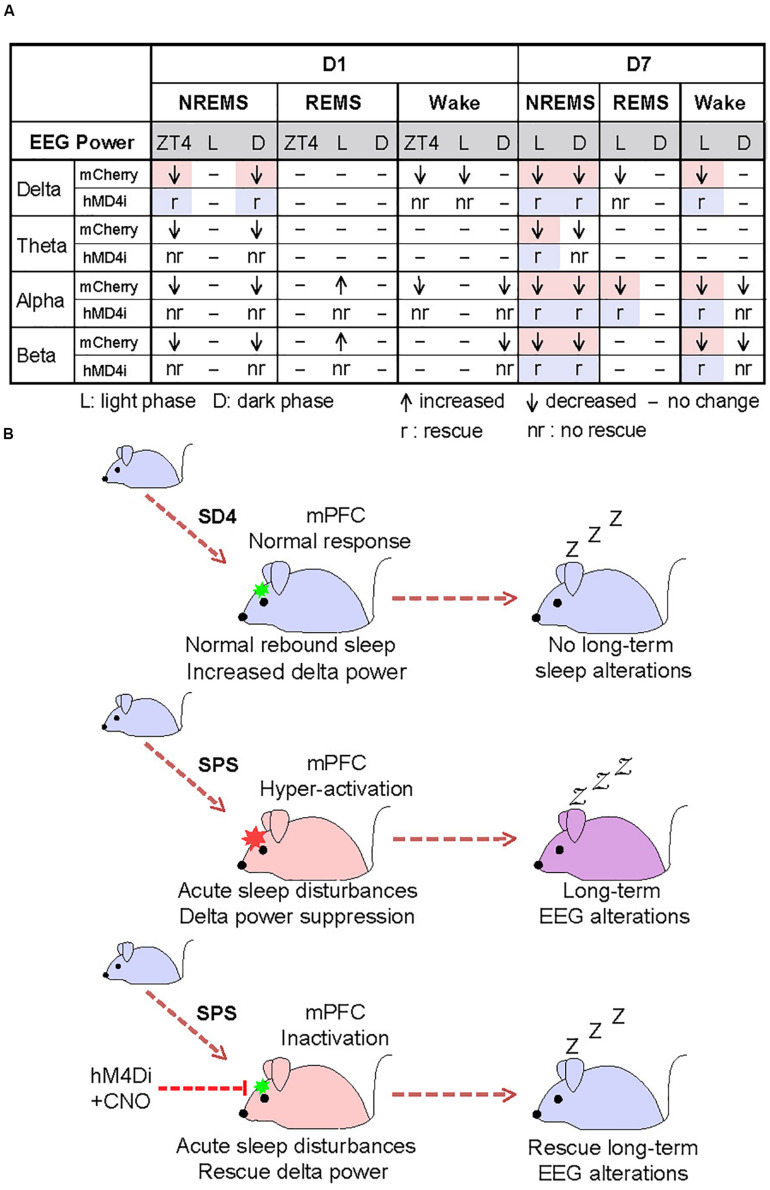
Hyper-activation of mPFC underlies traumatic stress-induced sleep–wake EEG disturbances. **(A)** A table summarizing the results of chemogenetic inhibition experiments. Highlighted regions indicate the specific SPS-induced short-term and long-term EEG abnormalities that can be rescued by chemogenetic inhibition of mPFC. **(B)** A model showing that hyper-activation of mPFC contributes critically to the SPS-induced sleep–wake EEG disturbances.

To test whether the hyper-activities of PrL neurons during SPS might also result in the traumatic stress-induced long-term sleep/wake EEG disturbances, we analyzed EEG/EMG data of the mCherry and hM4Di mice on the seventh day after SD4/SPS treatment (Figure 6A). Remarkably, we found that chemogenetic inhibition of PrL neurons could abrogate the majority of SPS-induced long-term sleep/wake EEG abnormalities on day 7 [Figures 6F, 7A and Supplementary Figure 7; (“SPS-D7” − “SD4-D7”)/“SD4-D7”: mCherry vs hM4Di]. Taken together, these results suggest that SPS-induced hyper-activation of mPFC neurons, particularly in the PrL region, may play a critical role in the development of both short- and long-term sleep–wake EEG disturbances (Figure 7B).

## Discussion

The sleep–wake disturbances may be one of the most debilitating symptoms associated with PTSD (Pawlyk et al., 2008). In this study, we adopted the well-established SPS paradigm to investigate the effects of traumatic stress on the sleep–wake architecture in the isogenic mouse model. In accordance with what Liberzon and colleagues had originally observed in SPS rats (Yamamoto et al., 2009), we showed that SPS mice also exhibited higher immobility time than control mice in the FST, but similar immobility time in the TST on the seventh day after SD4/SPS treatment (Supplementary Figure 4E). This result, together with our findings that SPS mice exhibited robust short and long-term sleep disturbances – a core symptom of PTSD patients – further validated the cross-species utility of mouse SPS-PTSD model. Because of the isogenic background and many genetics tools available, the mouse SPS-PTSD model offers unique advantages than the rat SPS-PTSD model in future mechanistic studies of traumatic stress-induced sleep disturbances.

The acute effects of SPS on sleep–wake architecture have been reported in two previous studies in rats (Nedelcovych et al., 2015; Vanderheyden et al., 2015). Our results are mostly consistent with earlier findings of Nedelcovych et al. (2015), but not those of Vanderheyden et al. (2015). A common finding of all three studies is the significant increase in REMS in the dark phase on the day after SPS (Figure 2). The importance of REMS rebound after acute stress is highlighted by the sleep assessment of humans who experience a traumatic event: those who exhibit long episodes of REMS do not develop PTSD, whereas those who have very short episodes of REMS are likely to develop PTSD (Mellman et al., 2002). Taken together, these findings suggest that REMS rebound during the first dark phase, especially long REMS episodes, may represent an essential adaptive strategy for animals or humans to cope with traumatic stress and avoid the development of PTSD (Stickgold, 2007).

We also observed a specific increase in absolute alpha and beta power of EEG signals during REMS and a broad reduction in absolute EEG power densities during NREMS and wake states after SPS (Figure 3). These significant changes in sleep/wake EEG power spectra may be attributed to traumatic stress-induced dys-regulation of multiple neuronal networks mediated by distinct neuromodulators (Vakalopoulos, 2014). It has been shown that traumatic stress causes serotonin release and regional utilization changes in multiple brain regions (Germain et al., 2008; Pawlyk et al., 2008; Nedelcovych et al., 2015). Several studies have also reported that acute stress increases acetylcholine release in the hippocampus and frontal cortex (Mark et al., 1996) and impairs signaling in the prefrontal cortex (Picciotto et al., 2012). These brain region-specific changes of neuromodulator signaling may lead to acute changes in sleep/wake duration and/or short- and long-term state-dependent EEG abnormalities.

Consistent with our previous studies (Funato et al., 2016; Wang et al., 2018), we found that absolute EEG power analysis could consistently outperform relative EEG power analysis by revealing more critical changes in the EEG power spectrum. Moreover, relative EEG power analysis could sometimes distort the data and reach the wrong conclusion (Figure 4E). Thus, we recommend that both absolute and relative EEG power analysis should be performed to obtain comprehensive phenotypic analysis in future patho/physiological sleep studies, especially when using a longitude experimental design in the isogenic mouse models.

Both acute and chronic stress can cause structural and functional alterations of the mPFC, resulting in dys-regulation of the cognitive-emotional control and threat extinction (Holmes and Wellman, 2009; Herringa, 2017; Heyn et al., 2019). Our chemogenetic inhibition experiments strongly suggest that the hyper-activation of mPFC neurons during SPS may mediate specific suppression of NREMS delta power immediately after SPS treatment (Figure 6), and eventually lead to the long-term sleep/wake EEG abnormalities (Figure 7). To our best knowledge, our study represents the first attempt to establish such a causality link between dysfunction of a specific brain region and traumatic stress-induced sleep/wake EEG abnormalities.

Recent studies suggest that the mPFC contains a heterogeneous neural population, including the pyramidal neurons and interneurons that may exert opposite regulation on EEG activities. Whereas pyramidal neuronal activity results in cortical activation and desynchronization, inhibitory interneurons that express somatostatin (SOM) are involved in the generation and propagation of slow waves characteristic of NREM sleep (Funk et al., 2017). Although the detailed mechanism by which the mPFC responds to SPS is unclear, we found that more than 95% of c-fos-expressing neurons in the mPFC are excitatory neurons (Figures 5E,F), suggesting that hyper-activities of pyramidal neurons, rather than interneurons such as SOM+ or parvalbumin+ interneurons, in the mPFC are probably involved in the acute suppression of NREMS delta power. However, future studies are needed to investigate the precise roles of different types of mPFC neurons in the SPS-induced sleep–wake EEG disturbances as the chemogenetic inhibition approach in our study result in the inhibition of all neuronal populations.

Both reduced and increased delta power activity during NREMS have been reported in PTSD patients (Woodward et al., 2000; Germain et al., 2006; Insana et al., 2012; de Boer et al., 2020; Wang et al., 2020). Thus, the SPS mouse model may recapitulate the symptoms of the subset of PTSD patients showing reduced NREMS delta power (Woodward et al., 2000; de Boer et al., 2020; Wang et al., 2020). In our study, we found that chemogenetic inhibition of the mFPC activity could specifically reverse the SPS-induced acute suppression of delta power during NREMS and most of the long-term sleep/wake EEG abnormalities. Moreover, sleep deprivation immediately after trauma, which normally elevates NREMS delta power during recovery sleep, has been reported as an effective intervention for attenuating PTSD-like behavioral disruptions (Cohen et al., 2012, 2017). These observations underscore the importance of sleep-dependent processes of neural reactivation in the development of PTSD (Cohen et al., 2012, 2017). Our findings may suggest the mPFC as an attractive target for the development of effective therapeutics for traumatic stress-induced psychiatric disorders, such as PTSD.

## Data Availability Statement

Source data and all other datasets generated and/or analyzed in the current study are available from the corresponding author on reasonable request.

## Ethics Statement

The animal study was reviewed and approved by the Institutional Animal Care and Use Committee of University of Tsukuba.

## Author Contributions

ZW, JM, and TL designed the experiments with technical assistances from YT, LC, C-YL, GA, and ZC. KS made the AAV virus. JM, TL, and ZW collected and analyzed the data. JM and ZW made the figures. ZW, QL, and KS wrote the manuscript. All authors contributed to the article and approved the submitted version.

## Conflict of Interest

The authors declare that the research was conducted in the absence of any commercial or financial relationships that could be construed as a potential conflict of interest.

## Supplementary Material

FIGURE S1 **(A)** Quantitative analysis of total NREMS, REMS, or wake time on the day before SD4/SPS treatment (SD4-BL vs SPS-BL). **(B,C)** Quantitative analysis of NREMS, REMS, or wake time on the day before and after 4 h sleep deprivation (SD4-D1 vs SD4-BL) **(B)**, and on the day before and after SPS treatment (SPS-D1 vs SPS-BL) **(C)**. *n* = 20, Mean ± s.d., paired *t*-test, two-tailed **(A–C)**. ^∗^*P* < 0.05; ^†^*P* < 0.01; ^‡^*P* < 0.001; ^ns^*P* > 0.05.

FIGURE S2 **(A–L)** Analysis of mean absolute EEG power density in every hour (left, hourly) or in the light/dark phase (right) in NREMS **(A–D)**, REMS **(E–H)** and wake **(I–L)** states of test mice (*n* = 20) on the day before SD4/SPS treatment (SD4-BL vs SPS-BL). Mean ± s.e.m., two-way ANOVA, Sidak’s test (for hourly analysis); Mean ± s.d., paired *t*-test, two-tailed (for mean analysis). ^∗^*P* < 0.05; ^†^*P* < 0.01; ^ns^*P* > 0.05.

FIGURE S3 **(A)** Analysis of mean absolute NREMS ZT4 EEG power density on the day before and after SD4/SPS treatment (SD4-D1 vs SD4-BL; SPS-D1 vs SPS-BL), (*n* = 20). **(B–D)** Analysis of mean absolute EEG power density in the light/dark phase during NREMS **(B)**, REMS **(C)** and wake **(D)** states of test mice (*n* = 20) on the day before and after SD4 treatment (SD4-D1 vs SD4-BL). Mean ± s.e.m., unpaired *t*-test, two-tailed **(A)**; Mean ± s.d., paired *t*-test, two-tailed **(B–D)**. ^∗^*P* < 0.05; ^†^*P* < 0.01; ^‡^*P* < 0.001; ^ns^*P* > 0.05.

FIGURE S4 **(A, B)** Hourly **(A)** and quantitative **(B)** analysis of NREMS, REMS and wake duration of test mice (*n* = 19) on day 7 after SD4 or SPS treatment (SD4-D7 vs SPS-D7). **(C,D)** Hourly analysis of episode duration **(C)** or episode number **(D)** of NREMS, REMS and wake states of test mice (*n* = 19) on D7 after SPS treatment (SD4-D7 vs SPS-D7). **(E)** Comparison of immobility time in the tail suspension test (TST) and forced swimming test (FST) on day 7 after SPS/SD4 treatment. SD4, *n* = 17; SPS, *n* = 12. Mean ± s.e.m., two-way RM ANOVA, Sidak’s test **(A,C,D)**; Mean ± s.d., paired *t*-test, two-tailed **(B)**; Mean ± s.e.m., unpaired *t*-test, two-tailed **(E)**. ^∗^*P* < 0.05; ^†^*P* < 0.01; ^‡^*P* < 0.001; ^ns^*P* > 0.05.

FIGURE S5 **(A,B)** Hourly **(A)** and quantitative **(B)** analysis of NREMS, REMS and wake duration of mCherry (*n* = 9) mice on the day after SD4/SPS treatment (SD4-D1 vs SPS-1). **(C,D)** Hourly **(C)** and quantitative **(D)** analysis of NREMS, REMS and wake duration of hM4Di (*n* = 9) mice on the day after SD4/SPS treatment (SD4-D1 vs SPS-D1). **(E)** Comparison of the difference (“SPS-D1” − “SD4-D1”) in NREMS, REMS or wake time of mCherry (*n* = 9) and hM4Di mice (*n* = 9) on the day after SD4/SPS treatment. Mean ± s.e.m., two-way RM ANOVA, Sidak’s test **(A,C)**; Mean ± s.d., paired *t*-test, two-tailed **(B,D)**; Mean ± s.e.m., unpaired *t*-test, two-tailed **(E)**. ^∗^*P* < 0.05; ^†^*P* < 0.01; ^‡^*P* < 0.001; ^ns^*P* > 0.05.

FIGURE S6 **(A–E)** Analysis of mean absolute NREMS **(A)**, REMS **(B)** or wake **(D)** EEG power density of mCherry (*n* = 9) and hM4Di (*n* = 9) mice on the day after SD4/SPS treatment (SD4-D1 vs SPS-D1). Comparison of the change ratio [(“SPS-D1” − “SD4-D1”)/”SD4-D1”]% in the mean absolute REMS **(C)** and wake **(E)** EEG power density of mCherry and hM4Di mice (*n* = 9) in the light or dark phase. Mean ± s.d., paired *t*-test, two-tailed **(A, B, D)**; Mean ± s.e.m., unpaired *t*-test, two-tailed **(C, E)**. ^∗^*P* < 0.05; ^†^*P* < 0.01; ^‡^*P* < 0.001; ^ns^*P* > 0.05.

FIGURE S7 **(A–E)** Analysis of mean absolute EEG power density during NREMS **(A)**, REMS **(B)** and wake **(D)** states of mCherry (*n* = 9) and hM4Di (*n* = 9) mice on day 7 after SD4/SPS treatment (SD4-D7 vs SPS-D7). Comparison of the change ratio [(“SPS-D7” − “SD4-D7”)/“SD4-D7”]% in the mean absolute EEG power density during REMS **(C)** and wake **(E)** states of mCherry (*n* = 9) and hM4Di (*n* = 9) mice in the light or dark phase on day 7 after SD4/SPS treatment. Mean ± s.d., paired *t*-test, two-tailed **(A,B,D)**; Mean ± s.e.m., unpaired *t*-test, two-tailed **(C,E)**. ^∗^*P* < 0.05; ^†^*P* < 0.01; ^‡^*P* < 0.001; ^ns^*P* > 0.05.

TABLE S1 Statistical analysis. The complete sample size, statistical test method, and precise value results for each comparison were reported.
