# Supplementary figures and images for: Hyper-Activation of mPFC Underlies Specific Traumatic Stress-Induced Sleep–Wake EEG Disturbances
Source: Front Neurosci. 2020 Aug 18;14:883. doi: 10.3389/fnins.2020.00883 (PMC7461881; doi:10.3389/fnins.2020.00883)

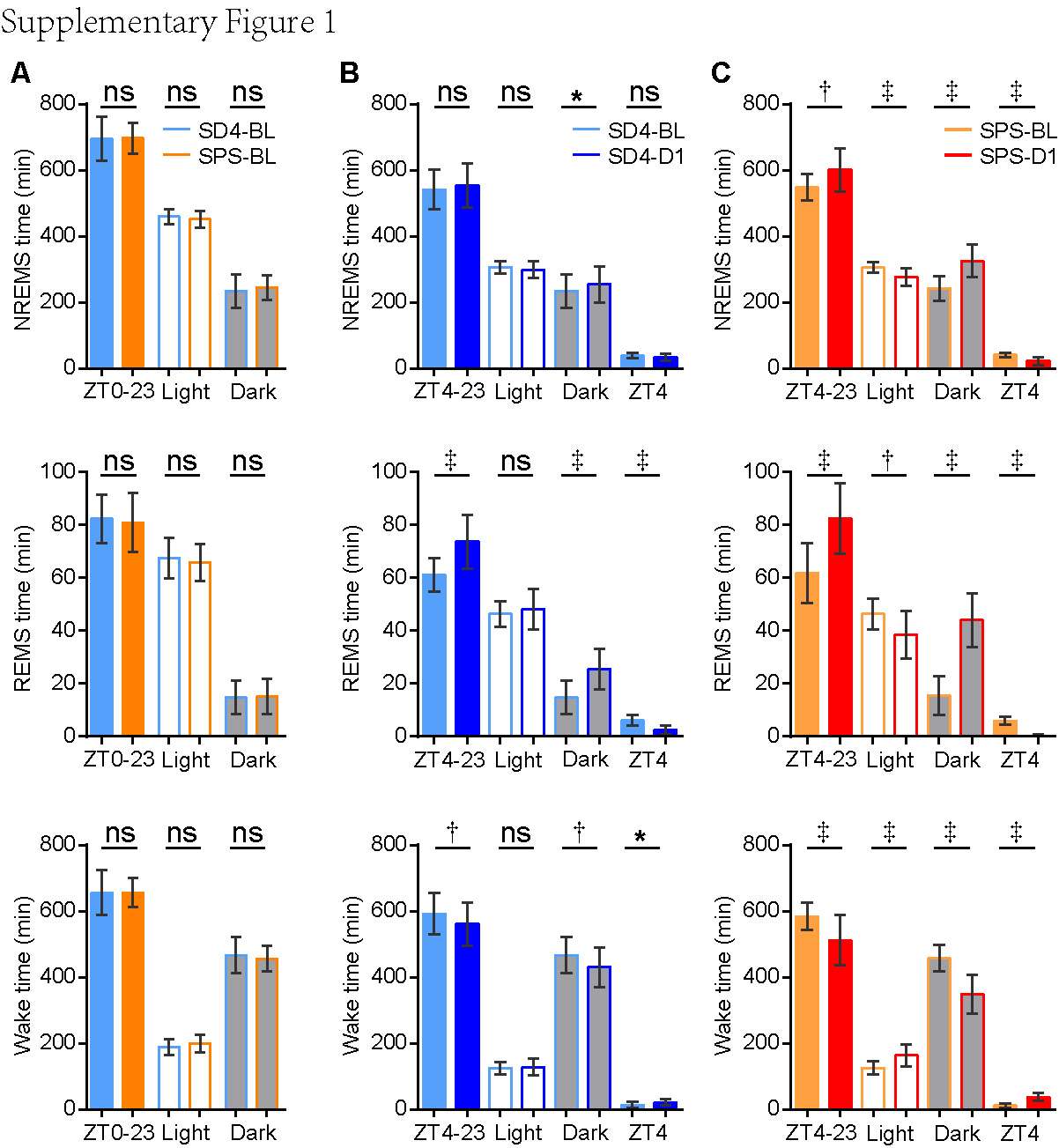

Supplement: FIGURE S1 — (A) Quantitative analysis of total NREMS, REMS, or wake time on the day before SD4/SPS treatment (SD4-BL vs SPS-BL). (B,C) Quantitative analysis of NREMS, REMS, or wake time on the day before and after 4 h sleep deprivation (SD4-D1 vs SD4-BL) (B), and on the day before and after SPS treatment (SPS-D1 vs SPS-BL) (C). n = 20, Mean ± s.d., paired t-test, two-tailed (A–C). ∗P < 0.05; †P < 0.01; ‡P < 0.001; nsP > 0.05. [file Image_1.JPEG]

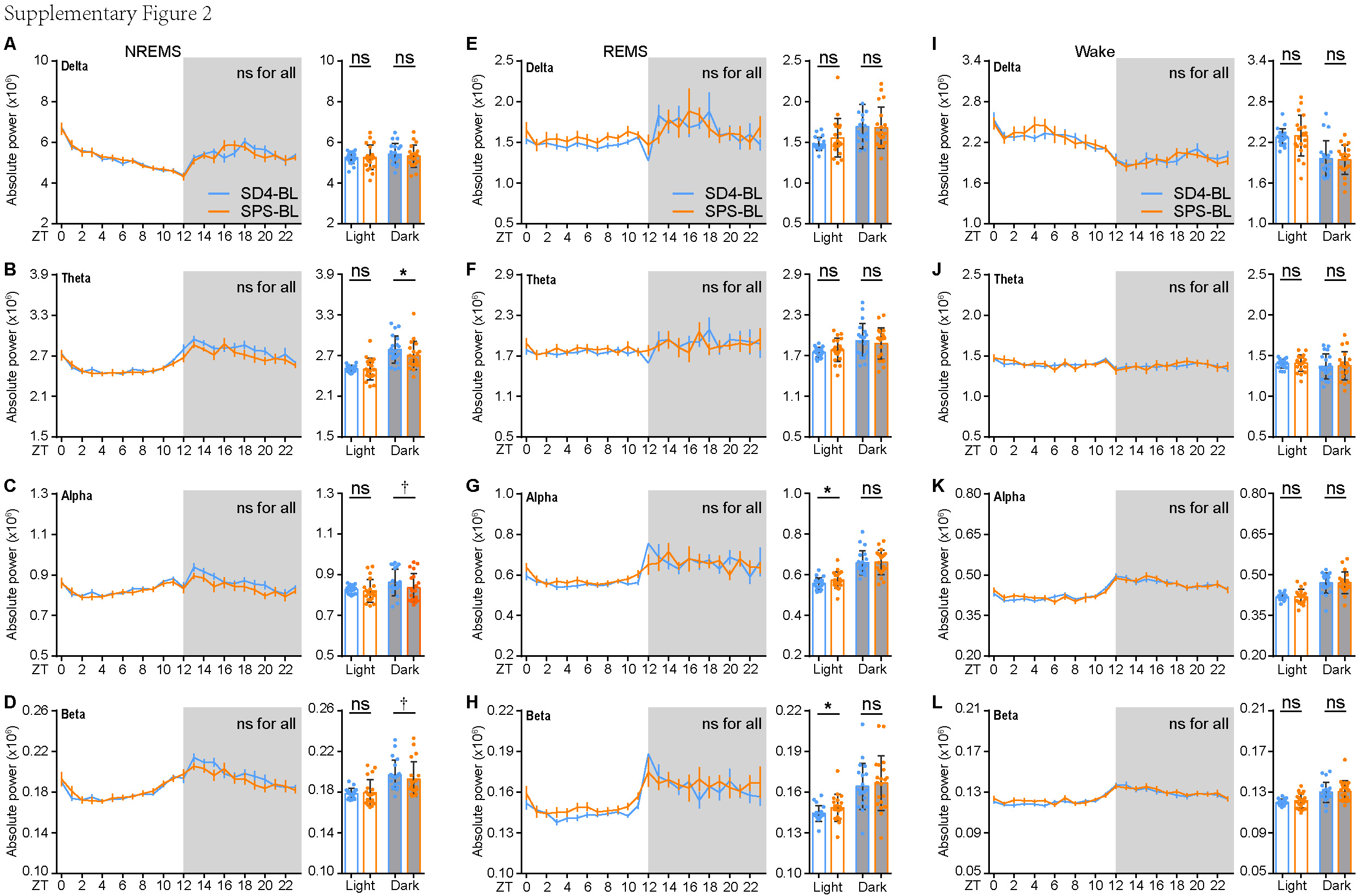

Supplement: FIGURE S2 — (A–L) Analysis of mean absolute EEG power density in every hour (left, hourly) or in the light/dark phase (right) in NREMS (A–D), REMS (E–H) and wake (I–L) states of test mice (n = 20) on the day before SD4/SPS treatment (SD4-BL vs SPS-BL). Mean ± s.e.m., two-way ANOVA, Sidak’s test (for hourly analysis); Mean ± s.d., paired t-test, two-tailed (for mean analysis). ∗P < 0.05; †P < 0.01; nsP > 0.05. [file Image_2.JPEG]

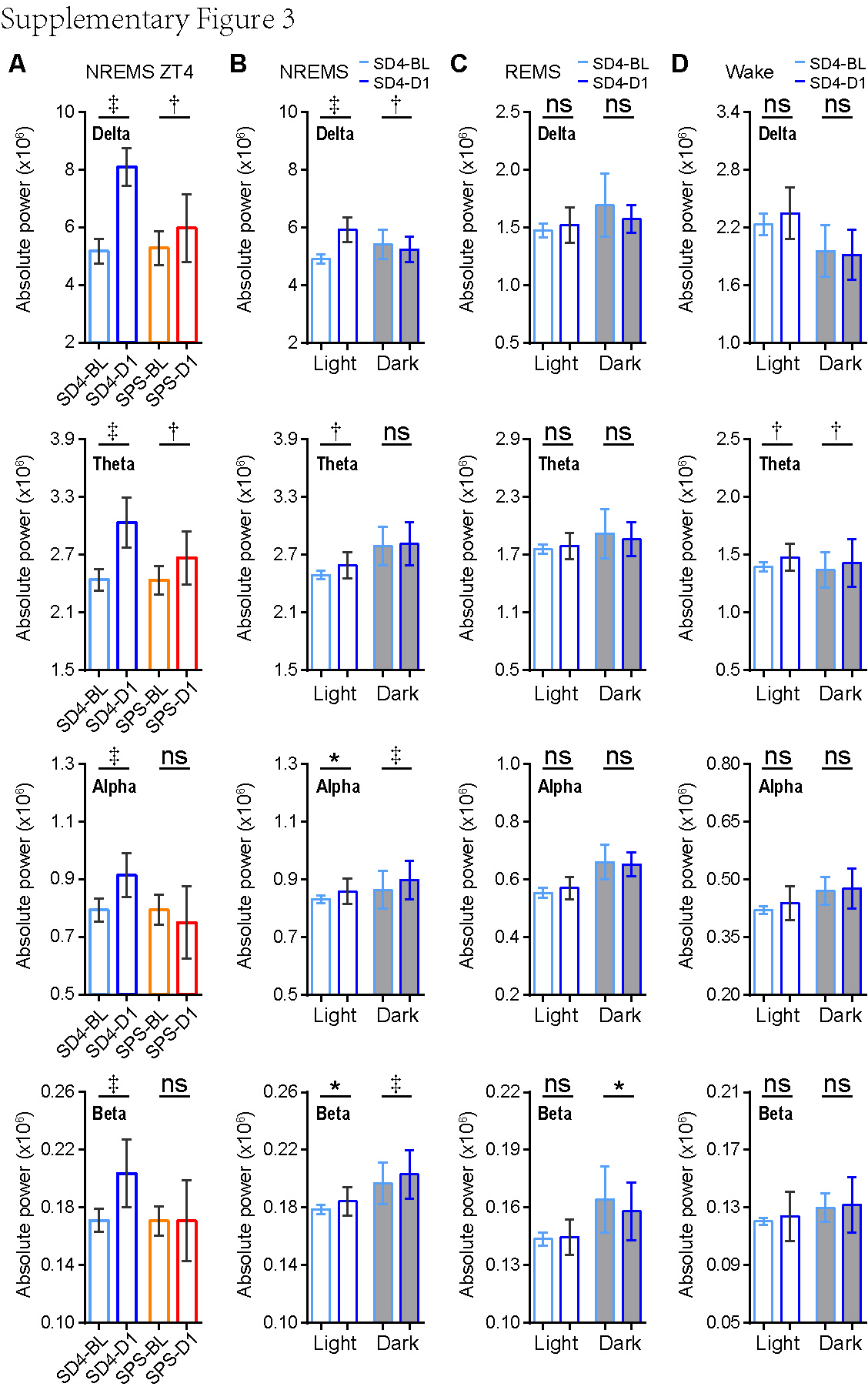

Supplement: FIGURE S3 — (A) Analysis of mean absolute NREMS ZT4 EEG power density on the day before and after SD4/SPS treatment (SD4-D1 vs SD4-BL; SPS-D1 vs SPS-BL), (n = 20). (B–D) Analysis of mean absolute EEG power density in the light/dark phase during NREMS (B), REMS (C) and wake (D) states of test mice (n = 20) on the day before and after SD4 treatment (SD4-D1 vs SD4-BL). Mean ± s.e.m., unpaired t-test, two-tailed (A); Mean ± s.d., paired t-test, two-tailed (B–D). ∗P < 0.05; †P < 0.01; ‡P < 0.001; nsP > 0.05. [file Image_3.JPEG]

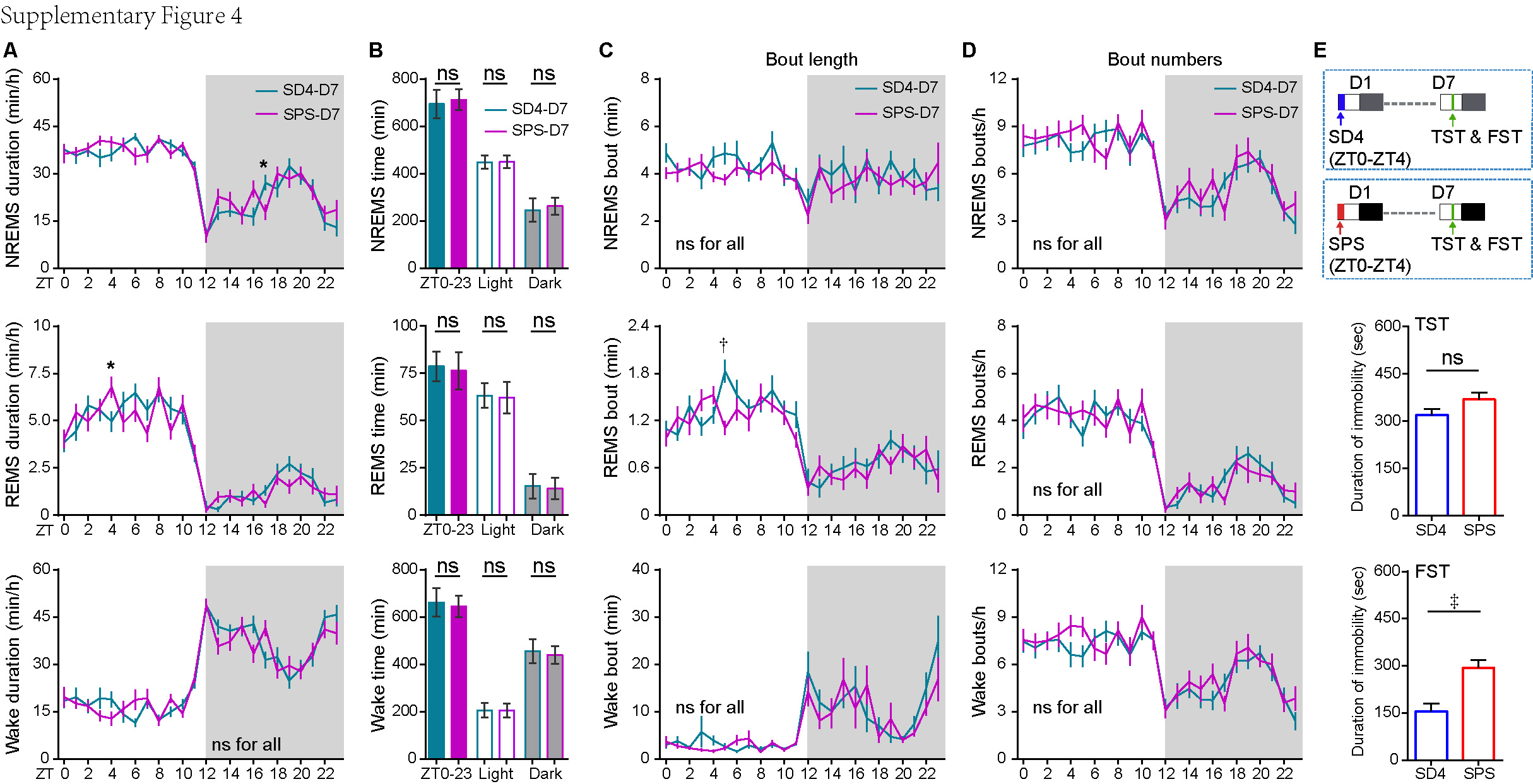

Supplement: FIGURE S4 — (A, B) Hourly (A) and quantitative (B) analysis of NREMS, REMS and wake duration of test mice (n = 19) on day 7 after SD4 or SPS treatment (SD4-D7 vs SPS-D7). (C,D) Hourly analysis of episode duration (C) or episode number (D) of NREMS, REMS and wake states of test mice (n = 19) on D7 after SPS treatment (SD4-D7 vs SPS-D7). (E) Comparison of immobility time in the tail suspension test (TST) and forced swimming test (FST) on day 7 after SPS/SD4 treatment. SD4, n = 17; SPS, n = 12. Mean ± s.e.m., two-way RM ANOVA, Sidak’s test (A,C,D); Mean ± s.d., paired t-test, two-tailed (B); Mean ± s.e.m., unpaired t-test, two-tailed (E). ∗P < 0.05; †P < 0.01; ‡P < 0.001; nsP > 0.05. [file Image_4.JPEG]

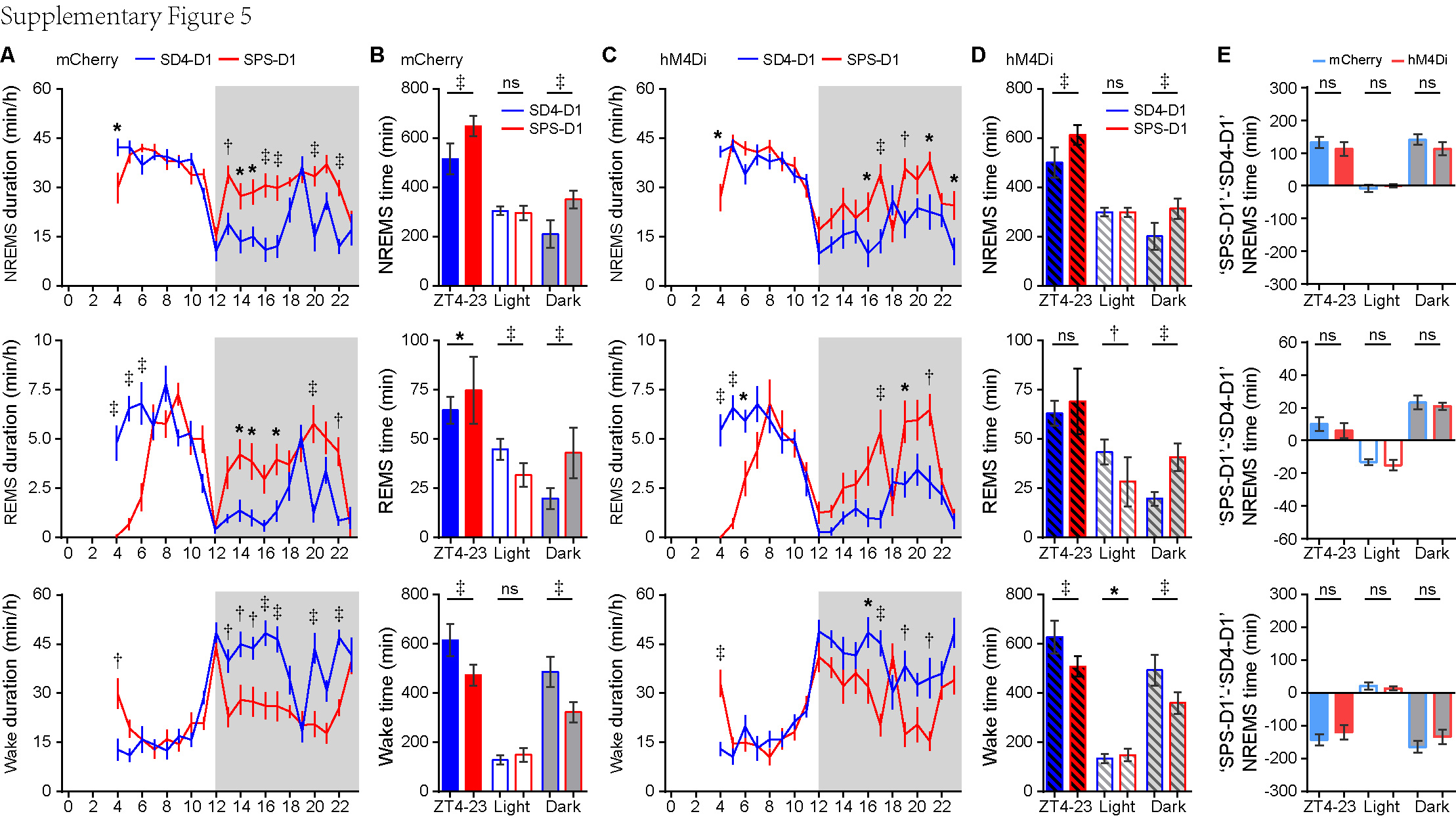

Supplement: FIGURE S5 — (A,B) Hourly (A) and quantitative (B) analysis of NREMS, REMS and wake duration of mCherry (n = 9) mice on the day after SD4/SPS treatment (SD4-D1 vs SPS-1). (C,D) Hourly (C) and quantitative (D) analysis of NREMS, REMS and wake duration of hM4Di (n = 9) mice on the day after SD4/SPS treatment (SD4-D1 vs SPS-D1). (E) Comparison of the difference (“SPS-D1” − “SD4-D1”) in NREMS, REMS or wake time of mCherry (n = 9) and hM4Di mice (n = 9) on the day after SD4/SPS treatment. Mean ± s.e.m., two-way RM ANOVA, Sidak’s test (A,C); Mean ± s.d., paired t-test, two-tailed (B,D); Mean ± s.e.m., unpaired t-test, two-tailed (E). ∗P < 0.05; †P < 0.01; ‡P < 0.001; nsP > 0.05. [file Image_5.JPEG]

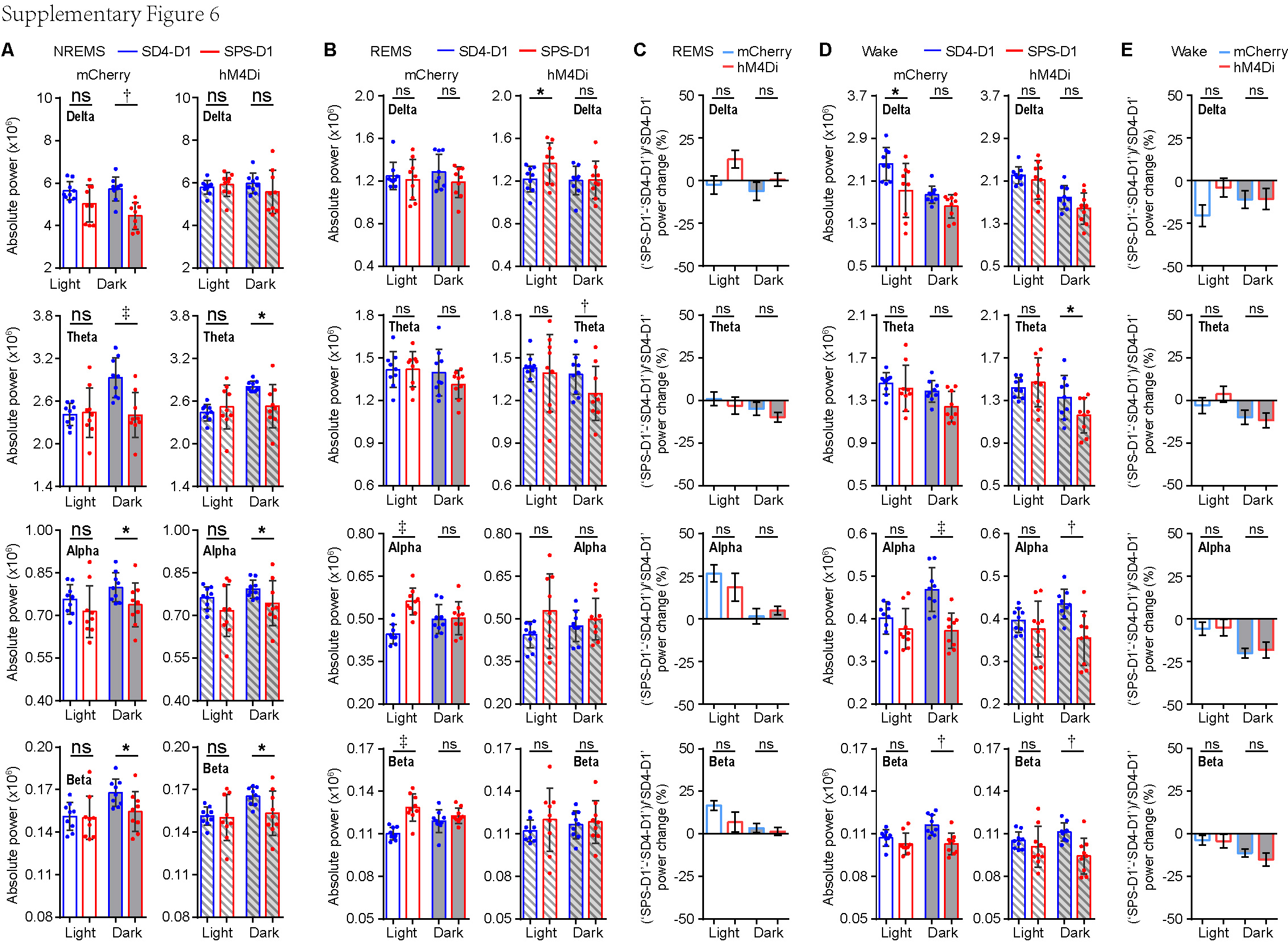

Supplement: FIGURE S6 — (A–E) Analysis of mean absolute NREMS (A), REMS (B) or wake (D) EEG power density of mCherry (n = 9) and hM4Di (n = 9) mice on the day after SD4/SPS treatment (SD4-D1 vs SPS-D1). Comparison of the change ratio [(“SPS-D1” − “SD4-D1”)/”SD4-D1”]% in the mean absolute REMS (C) and wake (E) EEG power density of mCherry and hM4Di mice (n = 9) in the light or dark phase. Mean ± s.d., paired t-test, two-tailed (A, B, D); Mean ± s.e.m., unpaired t-test, two-tailed (C, E). ∗P < 0.05; †P < 0.01; ‡P < 0.001; nsP > 0.05. [file Image_6.JPEG]

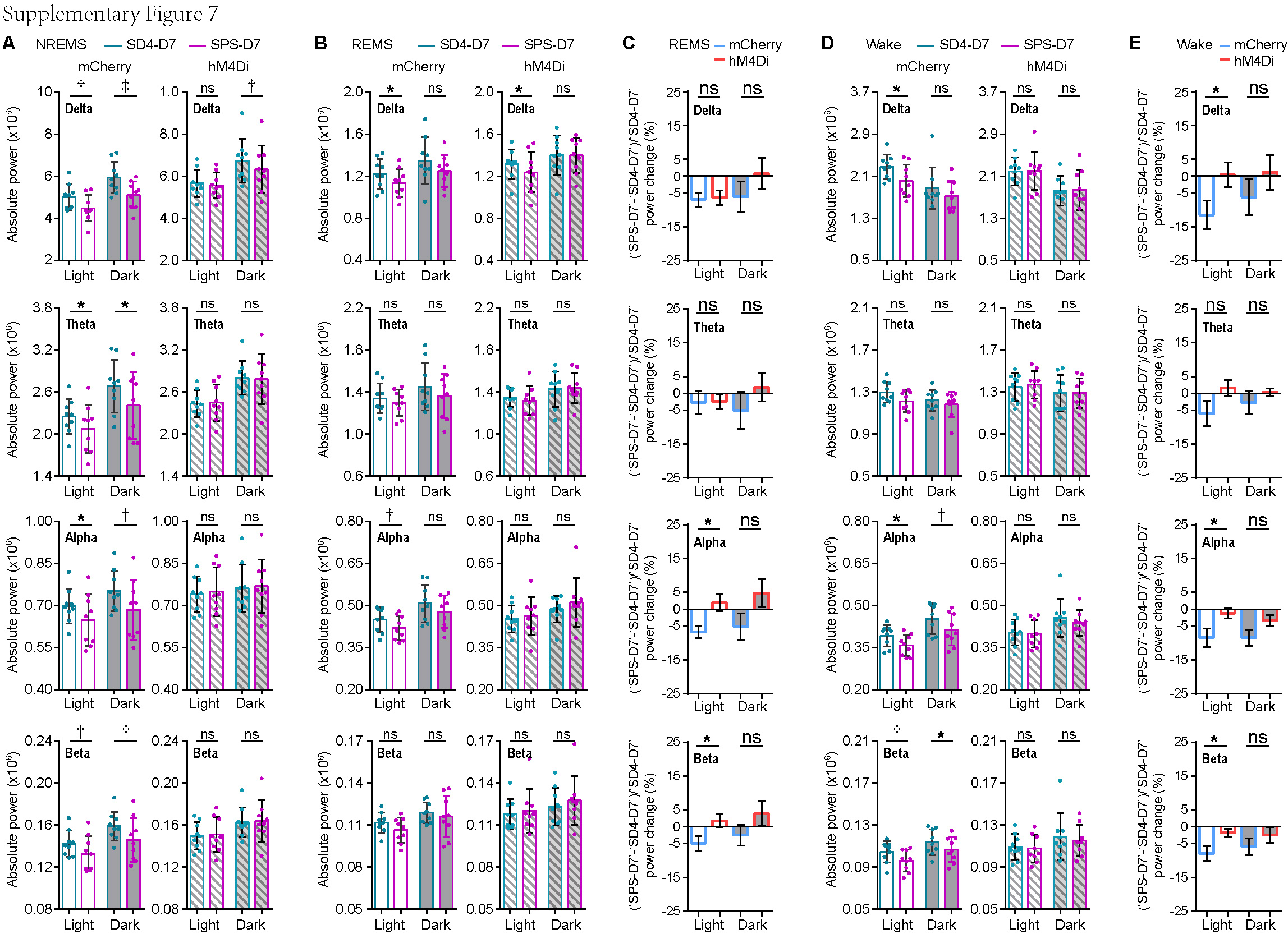

Supplement: FIGURE S7 — (A–E) Analysis of mean absolute EEG power density during NREMS (A), REMS (B) and wake (D) states of mCherry (n = 9) and hM4Di (n = 9) mice on day 7 after SD4/SPS treatment (SD4-D7 vs SPS-D7). Comparison of the change ratio [(“SPS-D7” − “SD4-D7”)/“SD4-D7”]% in the mean absolute EEG power density during REMS (C) and wake (E) states of mCherry (n = 9) and hM4Di (n = 9) mice in the light or dark phase on day 7 after SD4/SPS treatment. Mean ± s.d., paired t-test, two-tailed (A,B,D); Mean ± s.e.m., unpaired t-test, two-tailed (C,E). ∗P < 0.05; †P < 0.01; ‡P < 0.001; nsP > 0.05. [file Image_7.JPEG]
